# Supplementary material for: Using a pan-cancer atlas to investigate tumour associated macrophages as regulators of immunotherapy response
Source: Nat Commun. 2024 Jul 6;15:5665. doi: 10.1038/s41467-024-49885-8 (PMC11226649; doi:10.1038/s41467-024-49885-8)
Supplement: Supplementary file 1 — Supplementary Information [file 41467_2024_49885_MOESM1_ESM.pdf]

Using a pan-cancer atlas to investigate tumour-associated macrophages as regulators of immunotherapy response

2024-06-18

Contents

Supplementary Figures **2**

Supplementary Notes **12**

SN1. Macrophage communities exhibit marked polarization towards define assemblies 12

SN2. Further utilization of the atlas in a spatial context 15

Supplementary References **21**

Supplementary Figures

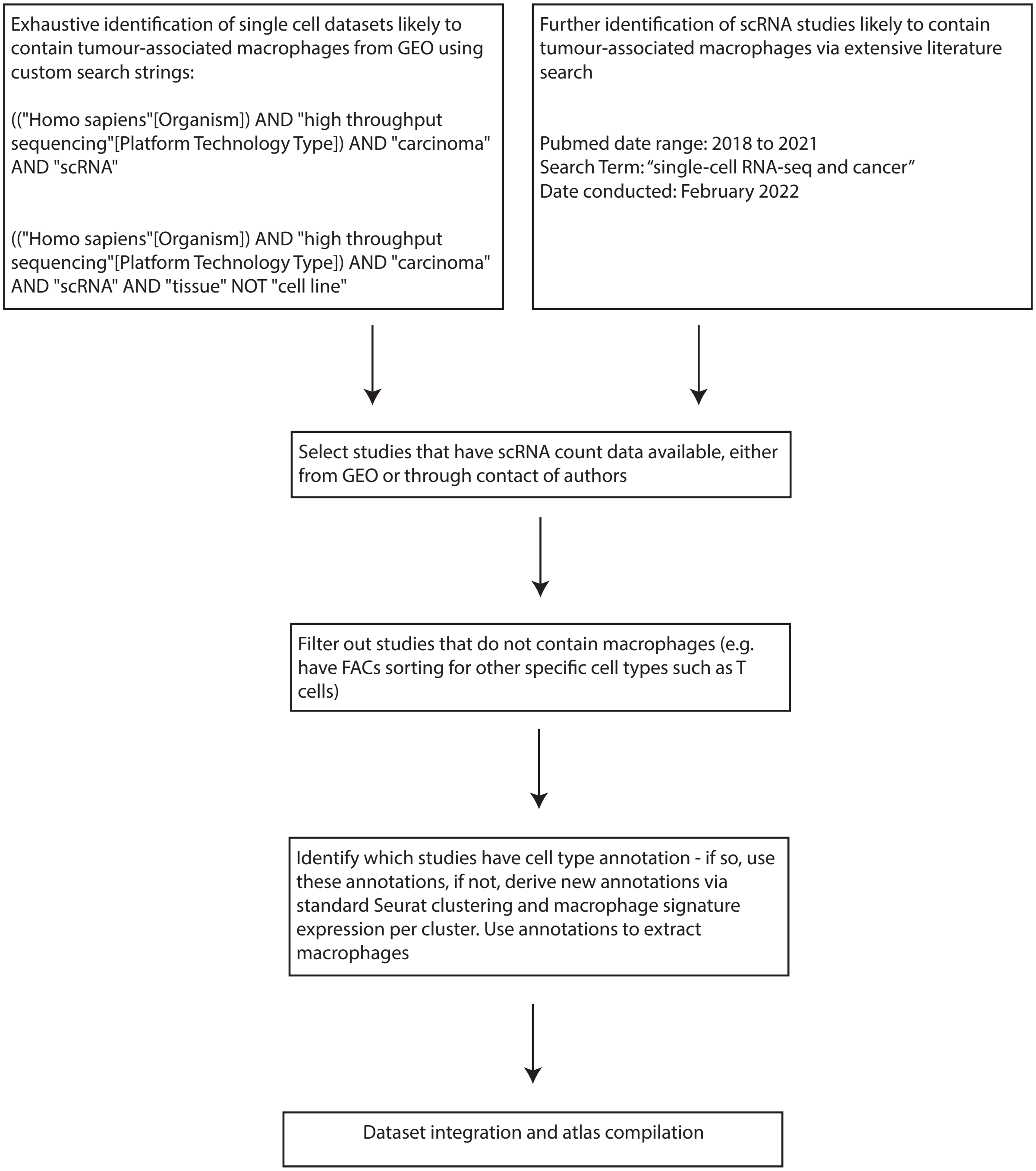

Supplementary Figure 1: Data acquisition strategy for compiling the atlas

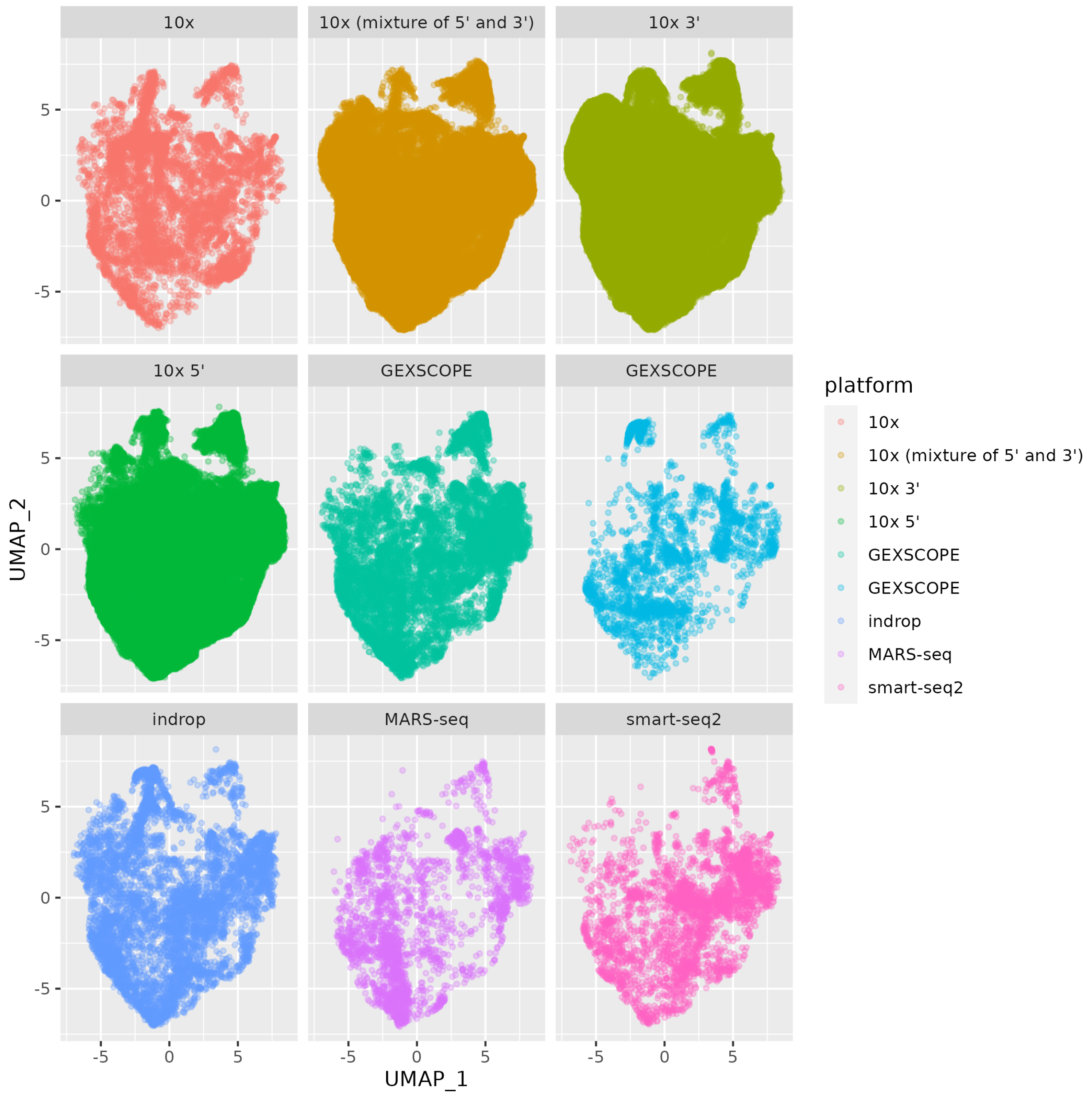

Supplementary Figure 2: UMAPs facetted by sequencing platform. None of the sequencing platforms exhibit strong biases towards a single cluster.

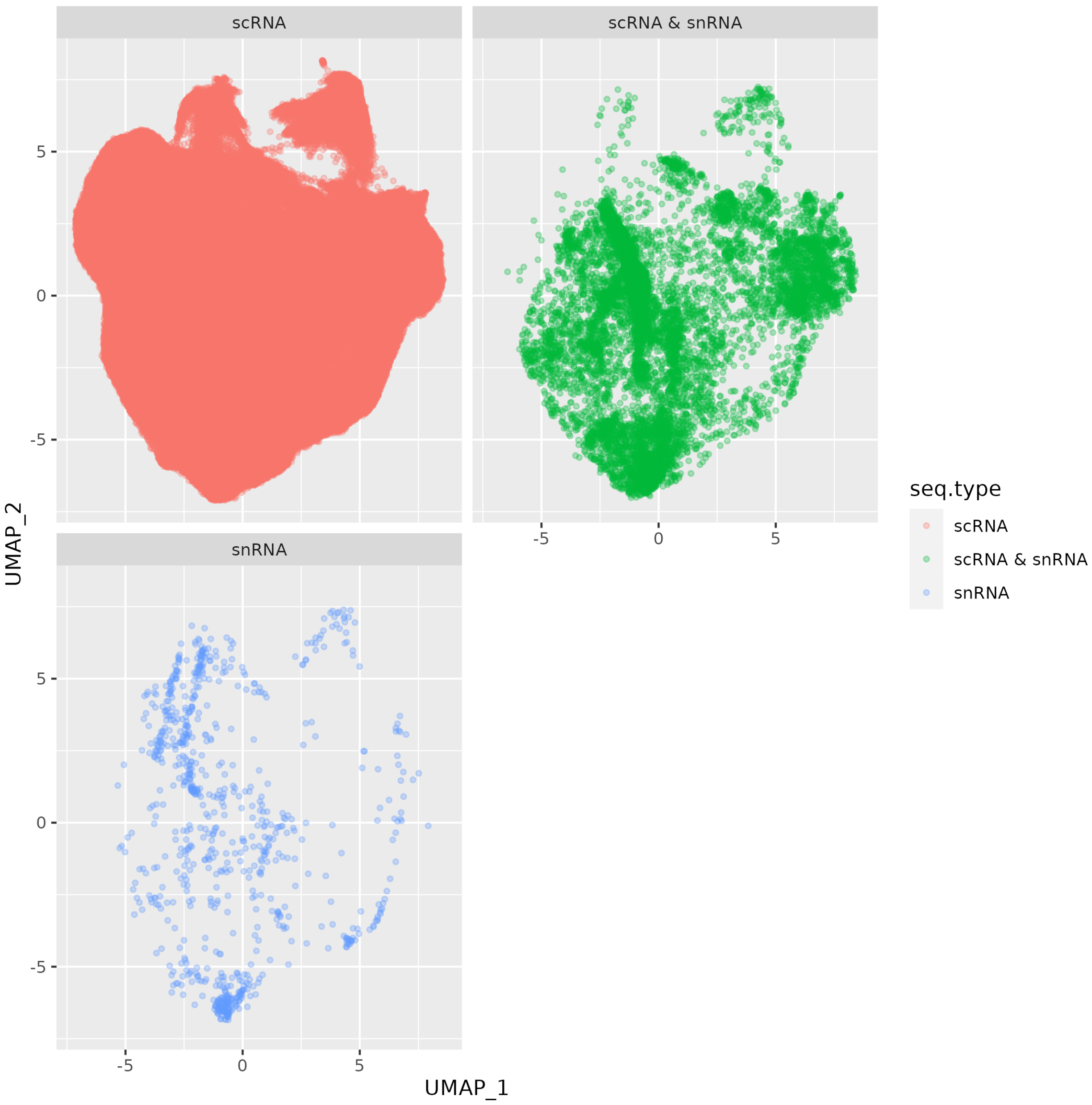

Supplementary Figure 3: UMAPs facetted by sequencing type (either scRNA, mixture of scRNA & snRNA, or snRNA). Cells in the snRNA study are distributed over several clusters.

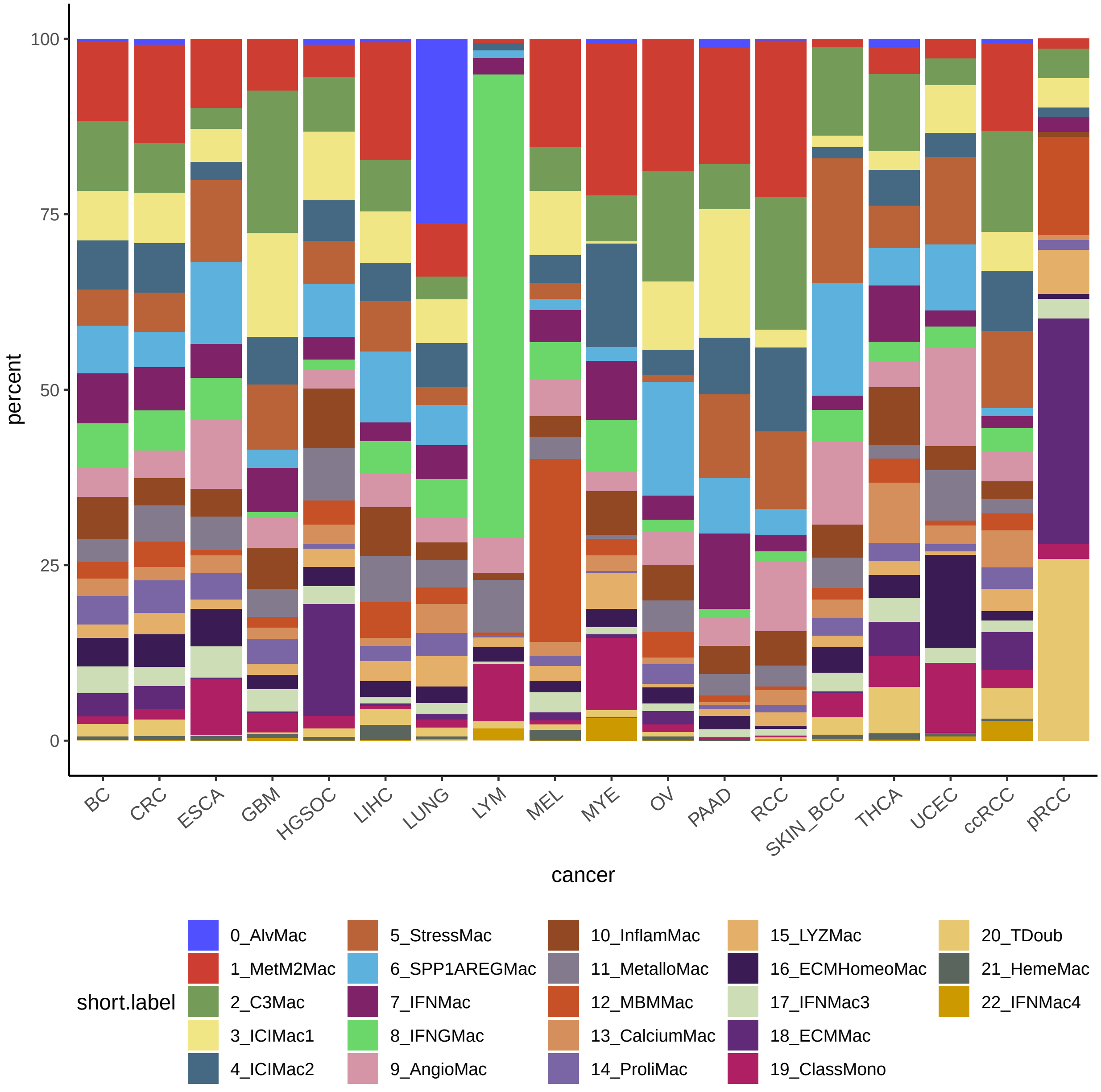

Supplementary Figure 4: Barplot showing the distribution of clusters for each cancer type.

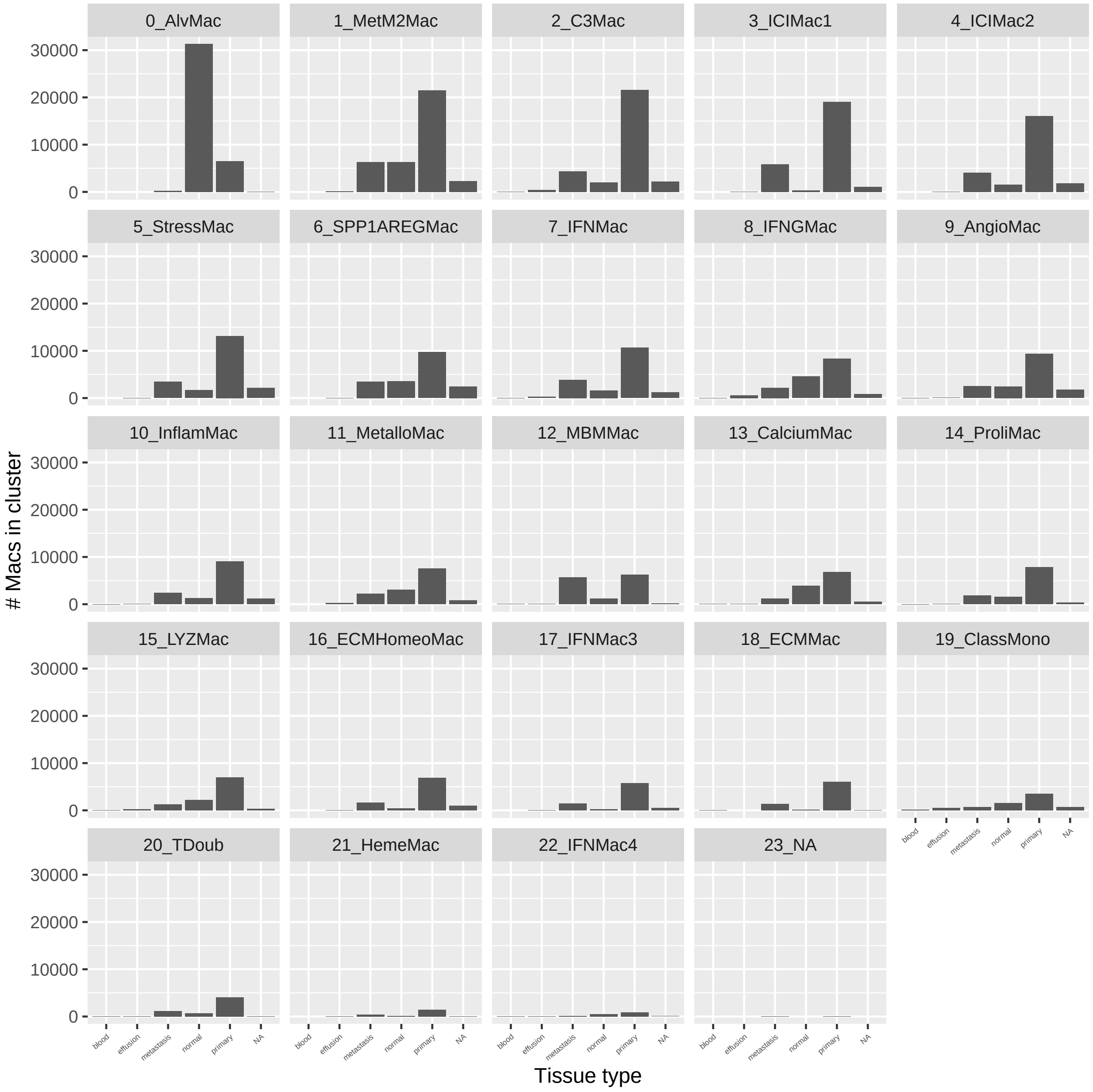

Supplementary Figure 5: Number of macrophages per tissue type facetted by cluster.

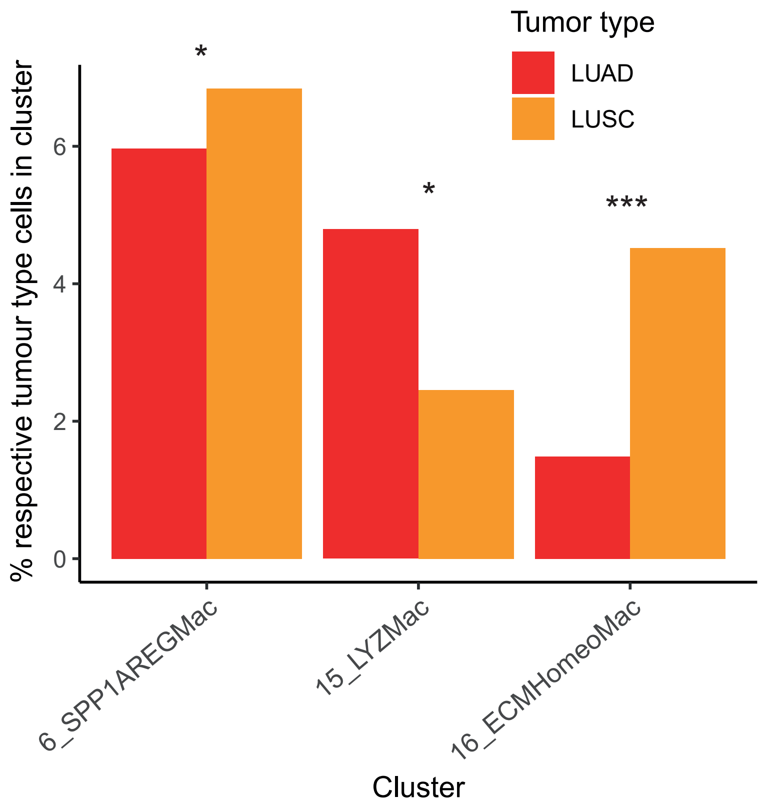

Supplementary Figure 6: Differences in cluster proportions between lung adenocarcinomas (LUAD) and lung squamous cell carcinomas (LUSC). Testing performed with moderated two-sided T-test via Propeller (1) with false-discovery rate correction for multiple testing. Only significantly different clusters between the two histologies as determined by Propeller, with FDR correction, are shown. * = q-value < 0.1, *** = q-value = 0.000008950575. n=7022, 5153, 2196 cells for 6_SPP1AREGMac, 15_LYZMac, 16_ECMHomeoMac respectively.

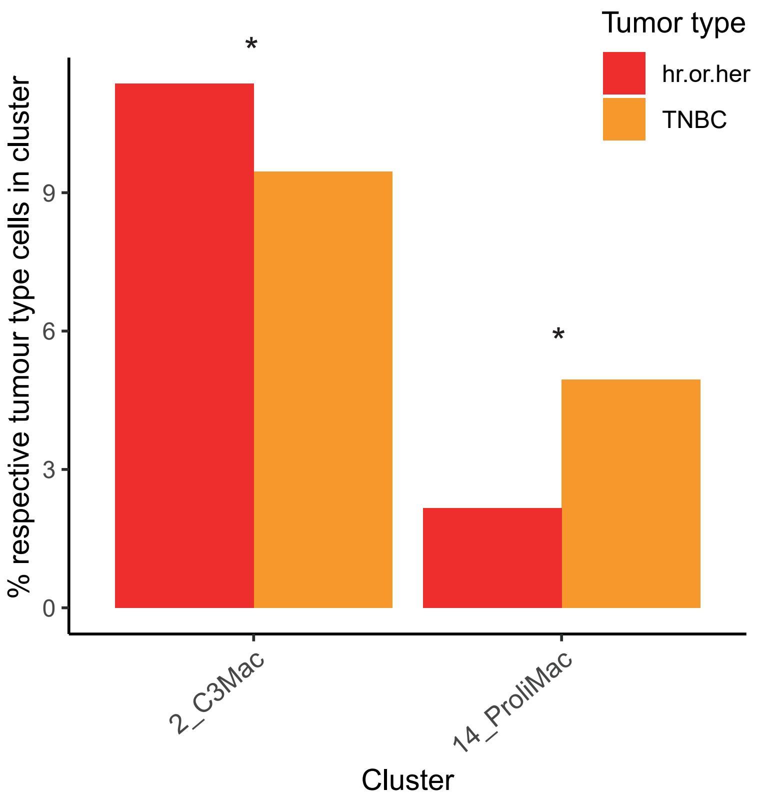

Supplementary Figure 7: Differences in cluster proportions between triple-negative breast cancers (TNBC) and HR or HER2 positive breast cancers (hr.or.her). Testing performed with moderated two-sided T-test via Propeller (1) with false-discovery rate correction for multiple testing. Only significantly different clusters between the two histologies as determined by Propeller, with FDR correction, are shown. * = q-value < 0.1. n=2549, 1073, cells for 3_C3Mac, 14_ProliMac respectively.

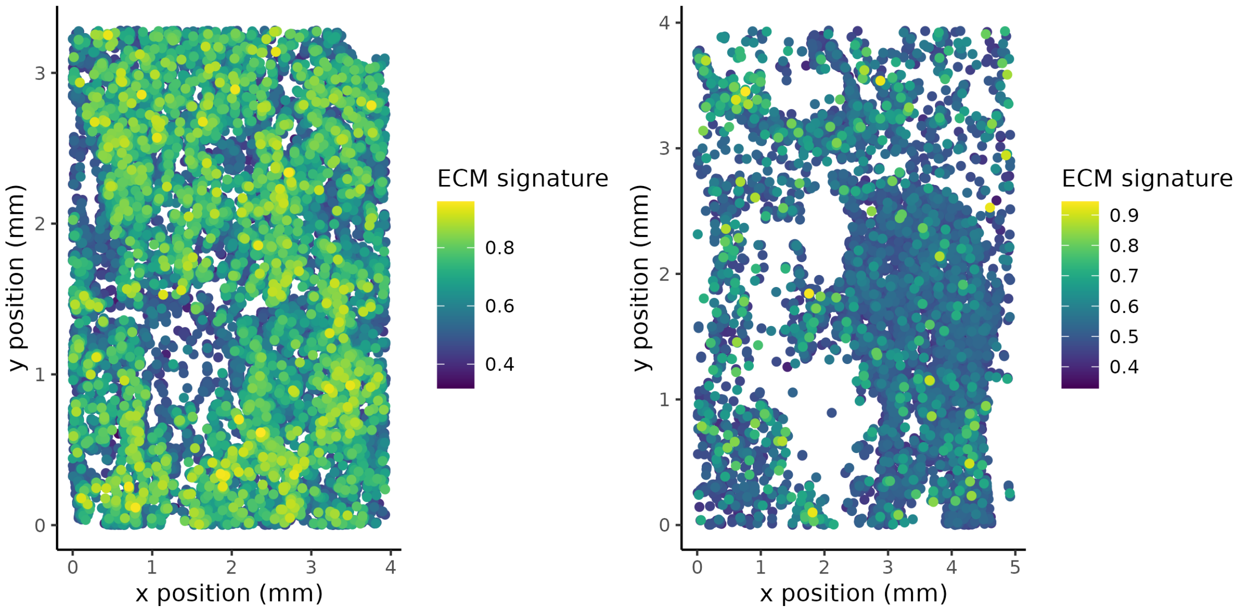

Supplementary Figure 8: We observed marked heterogeneity in ECM signature between different lung cancer tissue samples profiled with the CosMx platform. Colour represents UCell scores for the ECM signature per cell, whilst the x and y axes show the physical dimensions of the tissue slice.

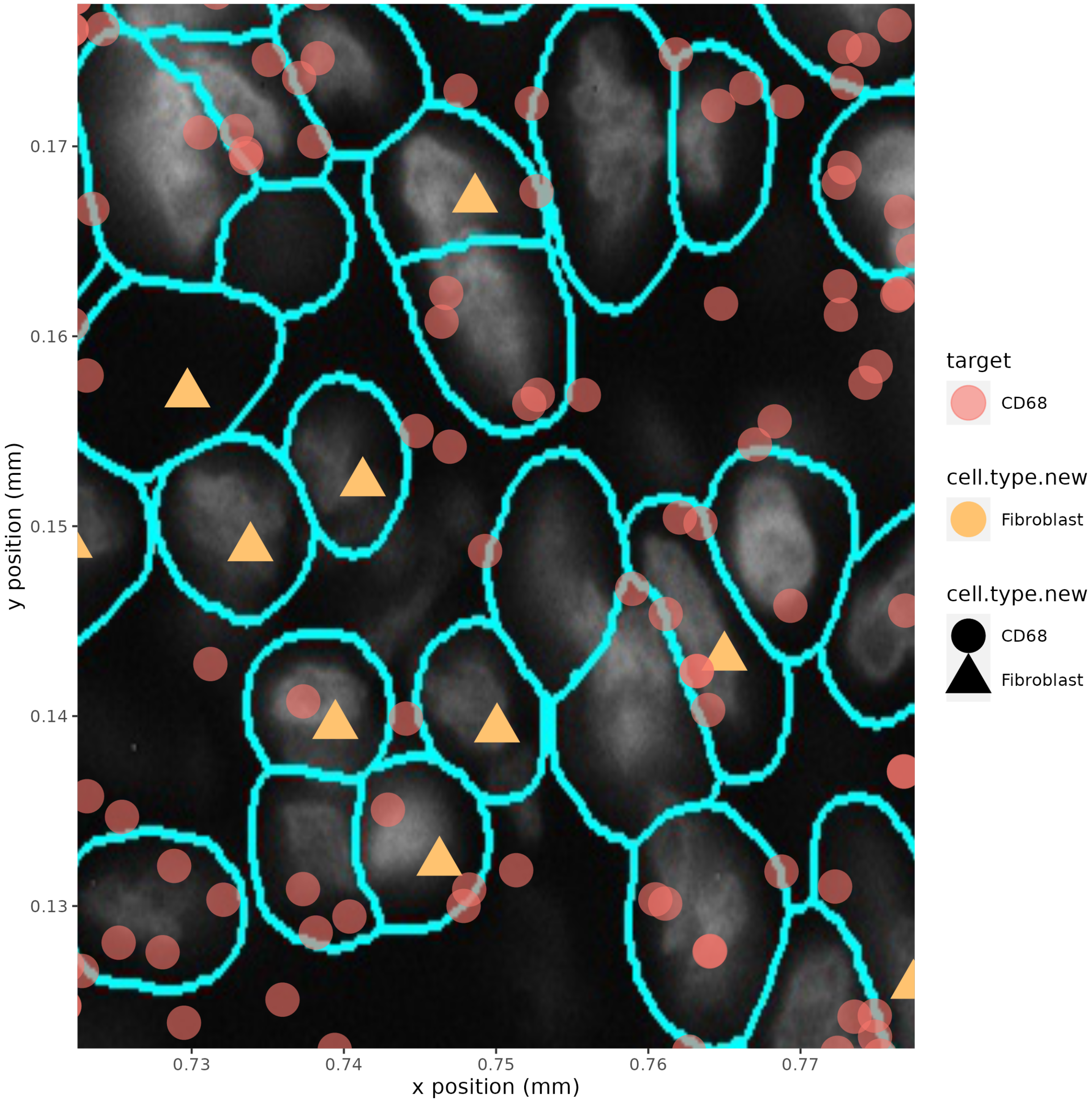

Supplementary Figure 9: Shown is a section of the Lung13 sample from the CosMx dataset. We observe several putative fibroblasts (yellow triangles), as identified by the CosMx cell type identification procedure detailed in He et al., 2022 (6), as expressing CD68 (red circles), the canonical macrophage marker. This is indicative of an intermediate macrophage-fibroblast cell state, that could potentially indicate differentiation between the two.

Supplementary Notes

SN1. Macrophage communities exhibit marked polarization towards define assemblies

Macrophage phenotype is influenced by a confluence of factors, including ontogeny, interactions with cytokines and chemokines in the environment, as well as interactions with neighboring cells. Tumours are complex environments containing many different cell types, with many tumours displaying heterogeneity in the cancer cells themselves, often with competing subclones under selective pressure from the immune system or drug treatment. Is this heterogeneity also present in the macrophage component of the tumour microenvironment, and if so, what is the degree of this heterogeneity within tumours? Existing studies have elucidated the ontological axis of this variation in select cancers, showing that the tumour-macrophage component changes with tumour progression, shifting from an assemblage of RTM-derived TAMs early in development to a predominance of monocyte-derived TAMs with tumour growth (2-3). Here we augment these studies, investigating this question in unprecedented detail using our large pan-cancer scRNAseq atlas.

We assessed the tendency of macrophage subsets to cooccur with each other using a pairwise correlation analysis of cluster membership within samples, revealing a diverse set of inter-cluster relationships. The clusters with the highest tendency to cooccur were 3_ICIMac1 and 16_ECMHomeoMac. TAMs in both of these clusters upregulate SPP1, whilst TAMs in the latter upregulate MMP9 and TIMP. SPP1, which encodes matricellular protein osteopontin, a non-structural ECM protein with regulatory functions (4), has been shown to induce the expression of MMP9, a matrix metalloproteinase, in fibroblasts in the context of Duchenne muscular dystrophy (5). The cooccurance of these clusters in our data suggest that this process also extends to macrophages. Also highly correlated were 10_InflamMac and 6_SPP1AREGMac. These two clusters are closely related (figure 2c), both of them primarily defined by their expression of a variety of cytokines, suggesting that perhaps these two clusters form two nearby points on a spectrum of differentiation. In addition, clusters 1_MetM2Mac and 4_ICIMac2 were also correlated in occurrence within samples. These clusters are both related to immunosuppression as discussed above, and their cooccurance suggests

that immunosuppression by macrophages operates on multiple modalities within tumours, rather than the singular “M2” categorization that is often applied.

In contrast, several subsets of macrophages were negatively correlated. The most negatively correlated subsets were 0_AlvMac and 22_IFNMac4, which is expected as 0_AlvMacs were primarily found in normal lung tissue, whereas 22_IFNMac4 appears to be a specialized subset of macrophages found in the ccRCC, with 67% of these macrophages originating from this cancer type. 0_AlvMac cells were also negatively correlated with 18_ECMMac cells, suggesting that 18_ECMMac is a phenotype that is initiated within tumours rather than normal tissue.

After examining the broad picture of macrophage co-occurrence, we sought to assess macrophage compositional diversity in more granular detail, applying an unsupervised learning approach to cluster samples based on their macrophage makeup. This method revealed a number of clear assemblages of clusters, spanning multiple samples, and often including several cancer types. These ranged from highly homogenous compositions characterized by polarization towards a single cluster, to more heterogenous compositions containing evenly distributed mixtures of macrophage subsets. Examples of the former (from left to right in Fig. 10d) include assemblages biased towards clusters 0_AlvMac, 3_ICIMac1, 12_MBMMac, 8_IFNGMac, 1_MetM2Mac, 2_C3Mac, 19_ClassMono and 5_StressMac. Some of the more heterogenous assemblages (right of Fig. 10d) were enriched for clusters 1_MetM2Mac and 2_C3Mac; 3_ICIMac1 and 7_IFNMac, as well as 1_MetM2Mac and 4_ICIMac2 respectively, as discussed above. CRC, MEL and ccRCC were represented broadly across most assemblages, whereas GBM, HGSOC and UCEC were more confined (Fig. 10c).

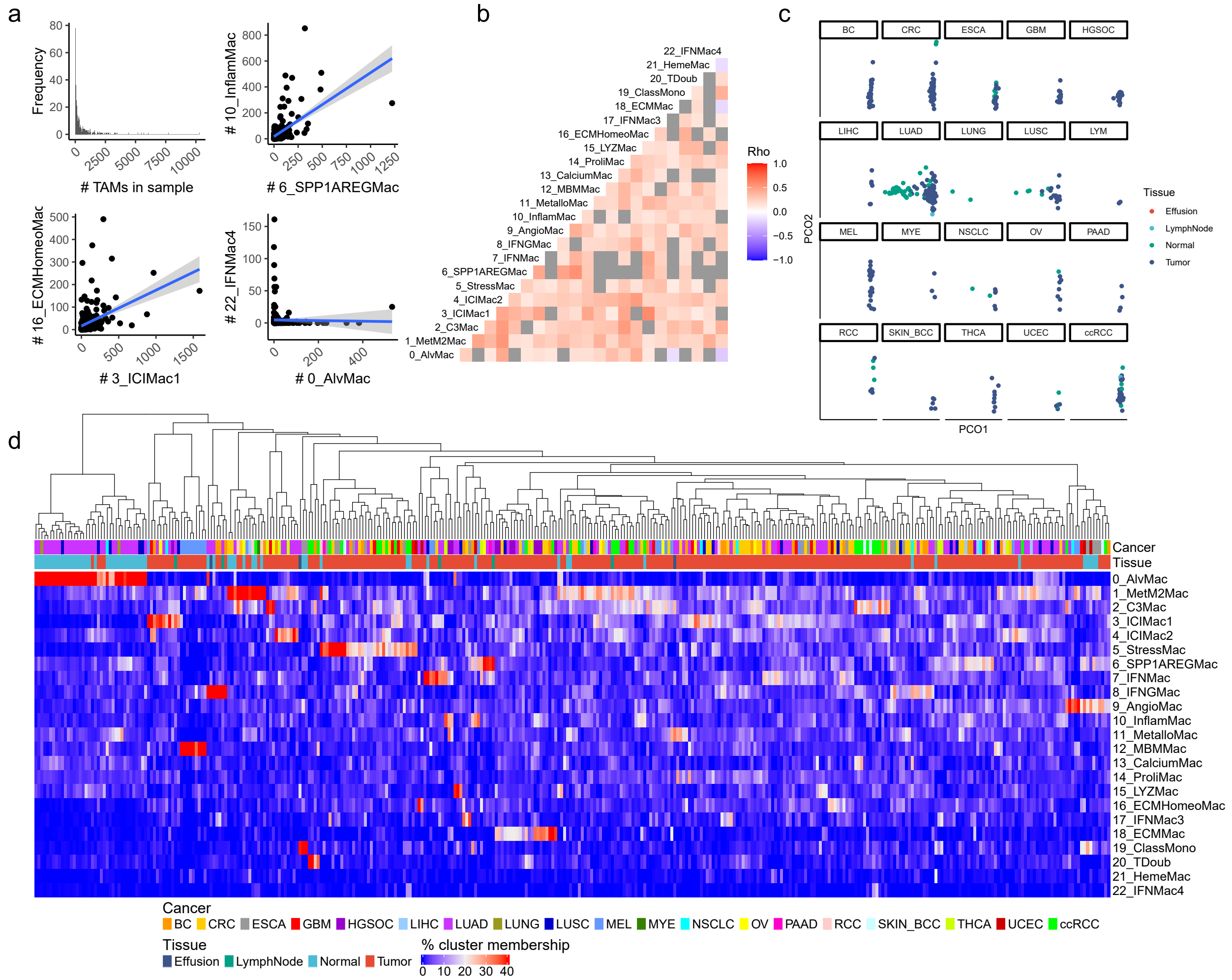

Supplementary Figure 10: Analysis of macrophage subset composition within samples. (a) Histogram showing number of TAMs per sample and scatterplots showing strongest positive / negative correlations in the pairwise analysis. (b) Pairwise correlation matrix showing subset co-occurance (red) or tendency towards mutual exclusivity (blue) of macrophage subsets within samples. Spearman's rho was used, and is indicated in colour, with non-significant correlations (q-value < 0.1 after false-discovery rate correction for multiple testing) indicated in grey. (c) PCO plots of sample similarity in terms of cluster composition facetted by cancer type. (d) Heatmap demonstrating macrophage composition types, with samples polarized towards one macrophage subset on the left, and more heterogenous samples on the right.

SN2. Further utilization of the atlas in a spatial context

In figure 5 of the manuscript, we demonstrate the utility of the atlas in classifying macrophages from novel datasets. Here we explore this further by examining an additional dataset (7) containing spatial RNASeq data. The Janesick study contains a number of single cell technologies, including Chromium 3' and 5' data from dissociated tumour cells, scFFPE-seq data from serial FFPE sections, as well as Visium and Xenium-processed samples from adjacent tissue sections to the FFPE sections to provide spatial insight. Visium contains 18085 genes at around ~ 50 cell resolution, whereas Xenium is a more select panel of 313 genes profiled at subcellular resolution.

We performed two analyses on this new data. The first was a projection of the cells labelled as macrophages by the authors on to our atlas, as performed previously on the oral cancer dataset of (8) in an attempt to classify them further. The original study did not perform extensive classification of macrophages, simply delineating them into two groups and listing top differentially expressed markers, so this analysis was useful in extending these results. The majority of macrophages were predicted to belong to the 1_MetM2Mac cluster (supp. Fig. 9; of which 8.66% are assigned to breast cancer in the atlas), followed by 12_MBMMac (4.96% assigned to breast cancer), and 3_ICIMac1 (7.36% assigned to breast cancer).

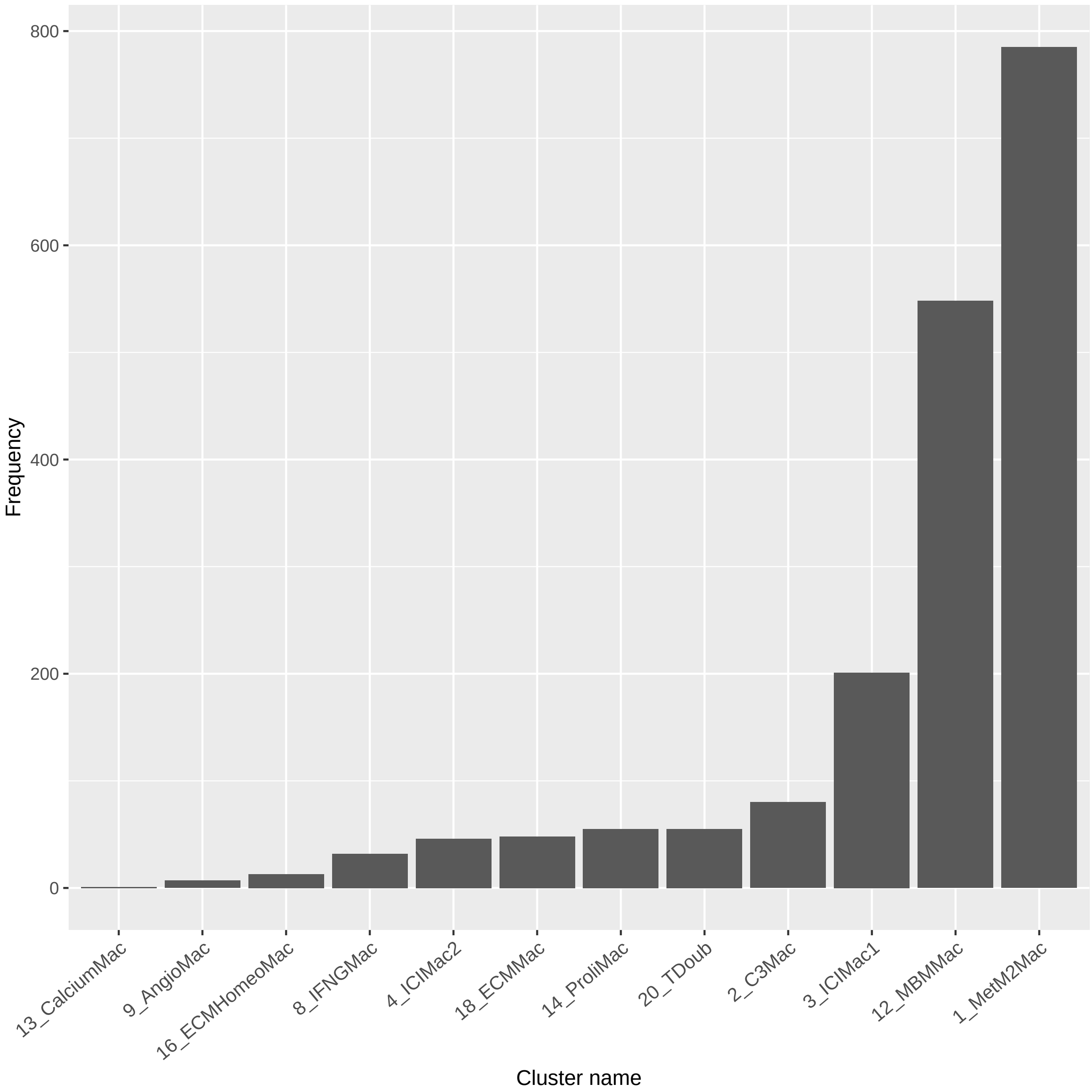

Supplementary Figure 11: Macrophage subtype predictions obtained from the single cell FFPE data, obtained through projection of cells classified as macrophages in the Janesick et al., 2023 (7) study on to our macrophage atlas.

Following our classification of the macrophages in the single cell FFPE data, we explored macrophages in a spatial context using the Visium data. To identify spots with likely presence of macrophages, we measured the geometric mean of each of our macrophage cluster signatures in the log-normalized data for each spot. Signature 1_MetM2Mac was most commonly the highest signature (supp. Fig. 10). Using all of the signatures instead of just 1_MetM2Mac served to validate our prediction from the initial projection analysis, as we would expect this signature to be highest. Following this, we visualized the 1_MetM2Mac signature across the tissue in a spatial manner (supp. Figs. 11-12). We found that regions annotated as adipocytes, stromal and mixed had the highest macrophage scores, whilst regions annotated as invasive, DCIS #1 and DCIS #2 has the lowest macrophage scores. We aimed to also analyse macrophages at cellular resolution using the Xenium data, but unfortunately the panel used for Xenium profiling did not contain a sufficient number of markers overlapping with the markers in our macrophage signatures.

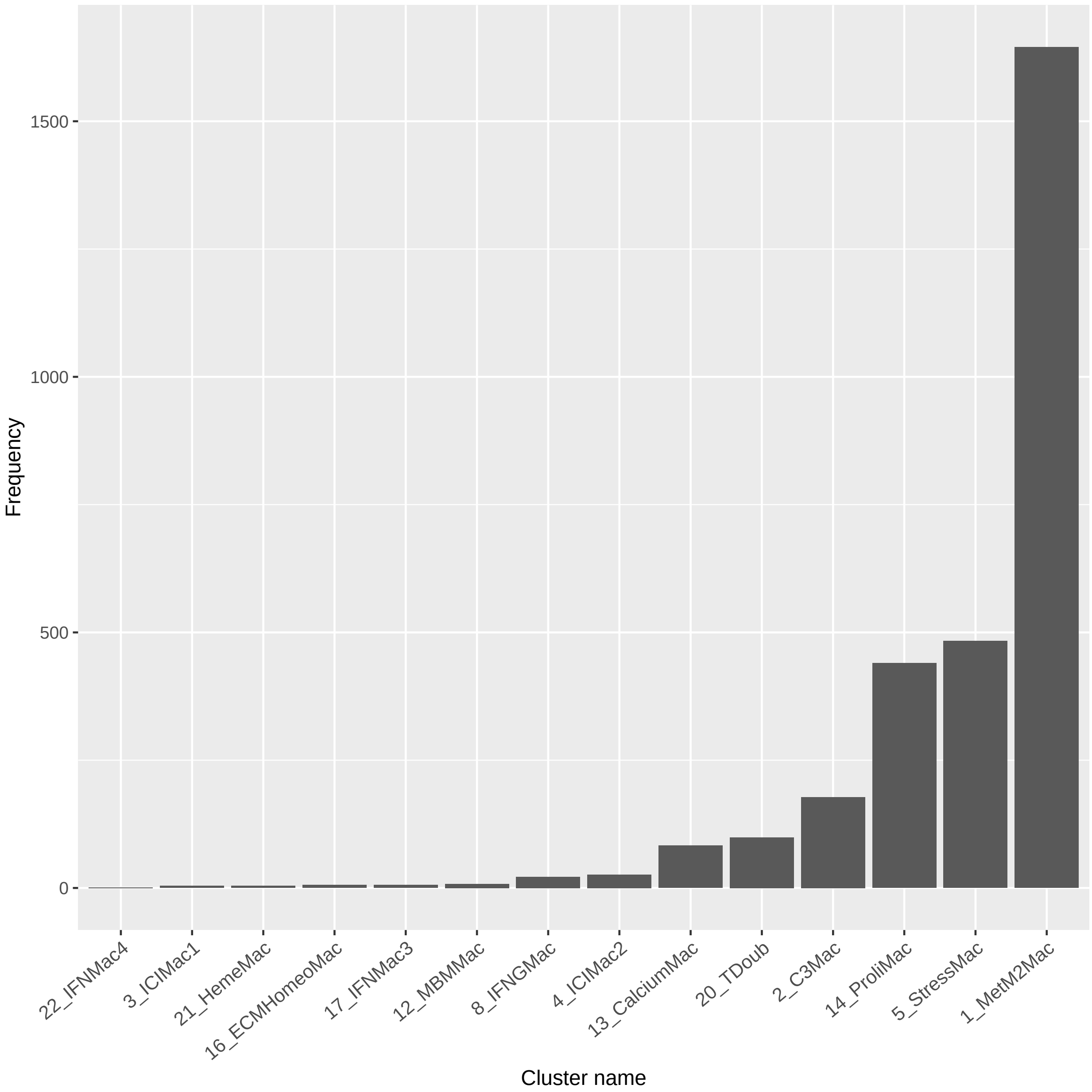

Supplementary Figure 12: Analysis of macrophage presence in the Visium data. For each spot, we measured the geometric mean of all of our macrophage signatures. Shown here are the frequencies of the signatures with the highest geometric mean in each spot.

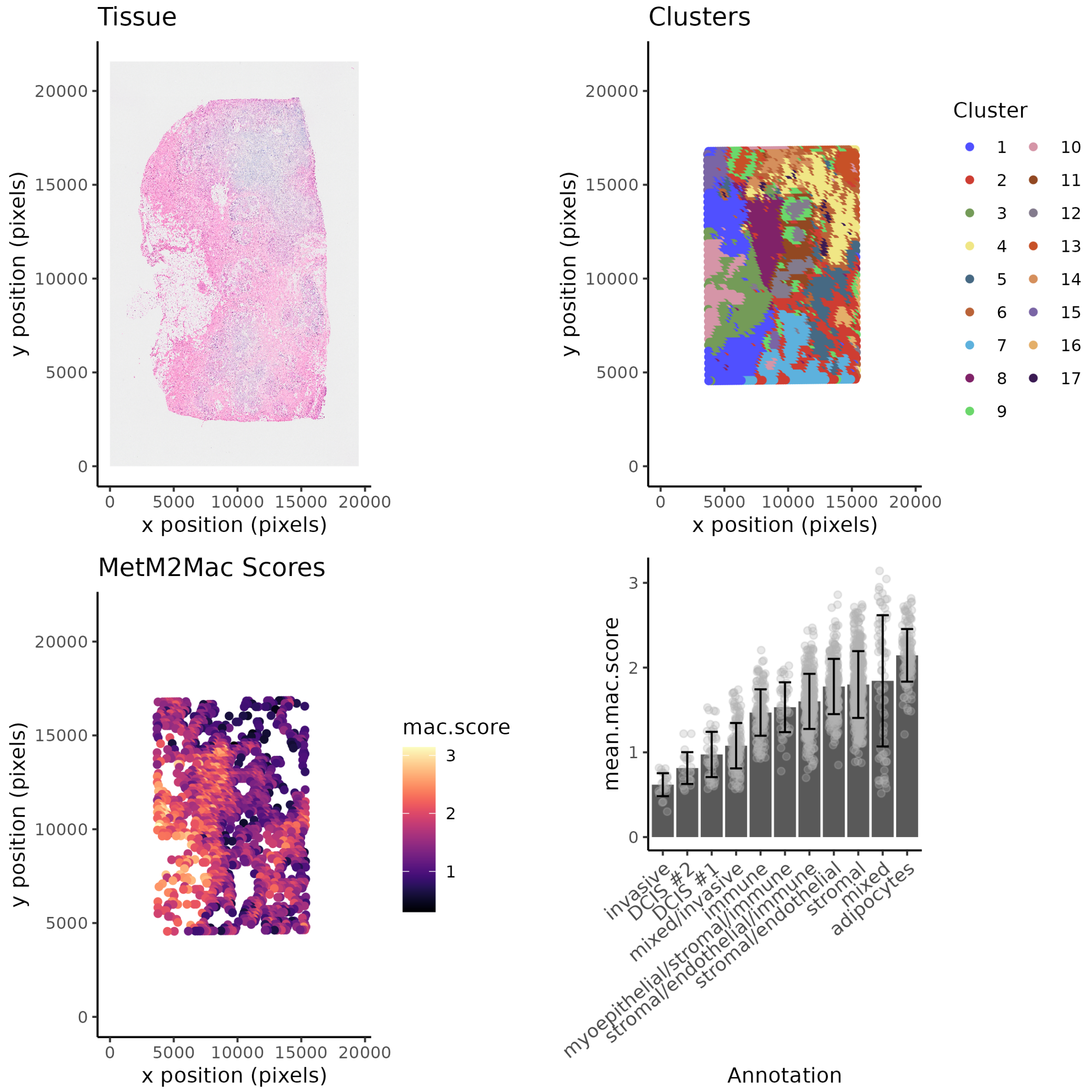

Supplementary Figure 13: Spatial visualization of macrophages in the Visium data. Top left: The breast cancer tissue under analysis. Top right: Clusters for each spot as assigned in the original Janesick et al., 2023 (7) data. Bottom left: 1_MetM2Mac signature scores in each spot (geometric mean). Bottom right: mean 1_MetM2Mac signature scores per annotation, annotations taken from the original study. Error bars represent ± standard deviation.

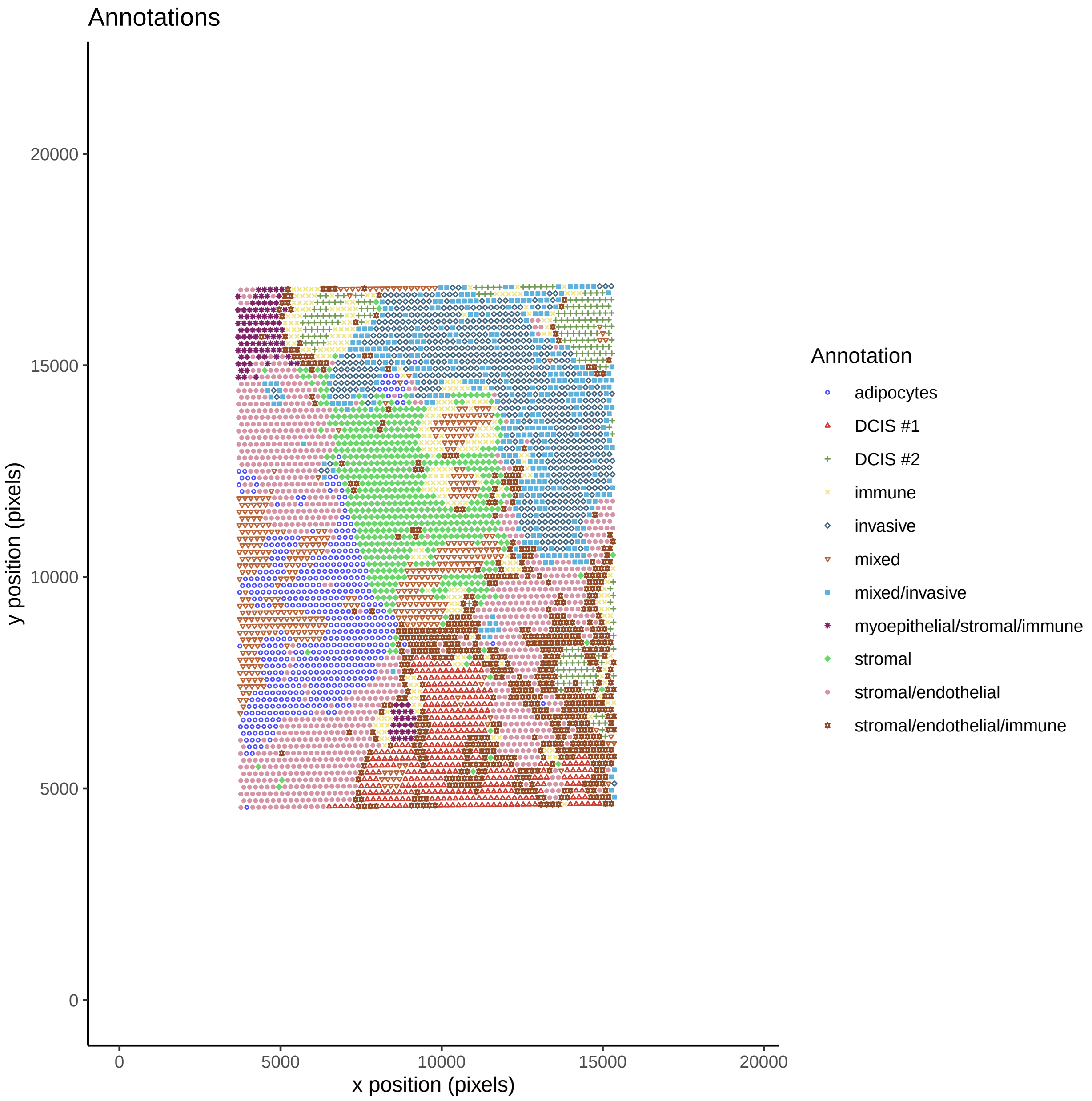

Supplementary Figure 14: Spatial visualization of spot annotations from Janesick et al., 2023 (7) paper.

Supplementary References

1. Phipson, B., Sim, C.B., Porrello, E.R., Hewitt, A.W., Powell, J., Oshlack, A., 2022. propeller: testing for differences in cell type proportions in single cell data. Bioinformatics 38, 4720–4726. https://doi.org/10.1093/bioinformatics/btac582

2. Casanova-Acebes, M., Dalla, E., Leader, A.M., LeBerichel, J., Nikolic, J., Morales, B.M., Brown, M., Chang, C., Troncoso, L., Chen, S.T., Sastre-Perona, A., Park, M.D., Tabachnikova, A., Dhainaut, M., Hamon, P., Maier, B., Sawai, C.M., Agulló-Pascual, E., Schober, M., Brown, B.D., Reizis, B., Marron, T., Kenigsberg, E., Moussion, C., Benaroch, P., Aguirre-Ghiso, J.A., Merad, M., 2021. Tissue-resident macrophages provide a pro-tumorigenic niche to early NSCLC cells. Nature 595, 578–584. https://doi.org/10.1038/s41586-021-03651-8

3. Franklin, R.A., Liao, W., Sarkar, A., Kim, M.V., Bivona, M.R., Liu, K., Pamer, E.G., Li, M.O., 2014. The cellular and molecular origin of tumor-associated macrophages. Science 344, 921–925. https://doi.org/10.1126/science.1252510

4. Singh, M., Foster, C.R., Dalal, S., Singh, K., 2010. Osteopontin: Role in extracellular matrix deposition and myocardial remodeling post-MI. Journal of Molecular and Cellular Cardiology, Special Issue: Extracellular Matrix 48, 538–543. https://doi.org/10.1016/j.yjmcc.2009.06.015

5. Kramerova, I., Kumagai-Cresse, C., Ermolova, N., Mokhonova, E., Marinov, M., Capote, J., Becerra, D., Quattrocelli, M., Crosbie, R.H., Welch, E., McNally, E.M., Spencer, M.J., 2019. Spp1 (osteopontin) promotes TGFβ processing in fibroblasts of dystrophin-deficient muscles through matrix metalloproteinases. Hum Mol Genet 28, 3431–3442. https://doi.org/10.1093/hmg/ddz181

6. He, S., Bhatt, R., Brown, C., Brown, E.A., Buhr, D.L., Chantranuvatana, K., Danaher, P., Dunaway, D., Garrison, R.G., Geiss, G., Gregory, M.T., Hoang, M.L., Khafizov, R., Killingbeck, E.E., Kim, D., Kim, T.K., Kim, Y., Klock, A., Korukonda, M., Kutchma, A., Lewis, Z.R., Liang, Y., Nelson, J.S., Ong, G.T., Perillo, E.P., Phan, J.C., Phan-Everson, T., Piazza, E., Rane, T., Reitz, Z., Rhodes, M., Rosenbloom, A., Ross, D., Sato, H., Wardhani, A.W., Williams-Wietzikoski, C.A., Wu, L., Beechem, J.M., 2022.

High-plex imaging of RNA and proteins at subcellular resolution in fixed tissue by spatial molecular imaging. Nat Biotechnol 40, 1794–1806. https://doi.org/10.1038/s41587-022-01483-z

7. Janesick, A., Shelansky, R., Gottscho, A.D., Wagner, F., Williams, S.R., Rouault, M., Beliakoff, G., Morrison, C.A., Oliveira, M.F., Sicherman, J.T., Kohlway, A., Abousoud, J., Drennon, T.Y., Mohabbat, S.H., Taylor, S.E.B., 2023. High resolution mapping of the tumor microenvironment using integrated single-cell, spatial and in situ analysis. Nat Commun 14, 8353. https://doi.org/10.1038/s41467-023-43458-x

8. Luoma, A.M., Suo, S., Wang, Y., Gunasti, L., Porter, C.B.M., Nabilsi, N., Tadros, J., Ferretti, A.P., Liao, S., Gurer, C., Chen, Y.-H., Criscitiello, S., Ricker, C.A., Dionne, D., Rozenblatt-Rosen, O., Uppaluri, R., Haddad, R.I., Ashenberg, O., Regev, A., Van Allen, E.M., MacBeath, G., Schoenfeld, J.D., Wucherpfennig, K.W., 2022. Tissue-resident memory and circulating T cells are early responders to pre-surgical cancer immunotherapy. Cell 185, 2918-2935.e29. https://doi.org/10.1016/j.cell.2022.06.018
