# Supplementary material for: Using a pan-cancer atlas to investigate tumour associated macrophages as regulators of immunotherapy response
Source: Nat Commun. 2024 Jul 6;15:5665. doi: 10.1038/s41467-024-49885-8 (PMC11226649; doi:10.1038/s41467-024-49885-8)
Supplement: Supplementary file 3 — Description of Additional Supplementary Files [file 41467_2024_49885_MOESM3_ESM.pdf]

Description of Additional Supplementary Files

Title: Supplementary Data 1

Description: Table detailing the sample type composition (primary, met, normal, blood) by cancer type

Title: Supplementary Data 2

Description: Table detailing the sample type composition (primary, metastasis, normal, blood) by study

Title: Supplementary Data 3

Description: Table detailing the number of macrophages and the number of non-macrophage cells for each study

Title: Supplementary Data 4

Description: Table detailing cluster composition by cancer type

Title: Supplementary Data 5

Description: Assessment of macrophage cluster signature performance with Ucell in an all celltype atlas. Columns show: the signature being assessed; the top hit cluster; the second best hit cluster; the cancer type being profiled; the mean Ucell score in the top hit cluster; the mean Ucell score in the second best hit cluster; the difference between these UCell scores; the mean difference between these UCell scores across all cancer types; the number of different clusters in the best hit category across the 5 cancer types being assessed; "gold-standard" designation respectively.

Title: Supplementary Data 6

Description: Table containing atlas meta data.
