# Supplementary material for: Using a pan-cancer atlas to investigate tumour associated macrophages as regulators of immunotherapy response
Source: Nat Commun. 2024 Jul 6;15:5665. doi: 10.1038/s41467-024-49885-8 (PMC11226649; doi:10.1038/s41467-024-49885-8)
Supplement: Supplementary file 6 — Reporting Summary [file 41467_2024_49885_MOESM6_ESM.pdf]

nature portfolio

Corresponding author(s): Dr Kevin Litchfield; Professor Claire Lewis

Last updated by author(s): May 28, 2024

Reporting Summary

Nature Portfolio wishes to improve the reproducibility of the work that we publish. This form provides structure for consistency and transparency in reporting. For further information on Nature Portfolio policies, see our Editorial Policies and the Editorial Policy Checklist.

Statistics

For all statistical analyses, confirm that the following items are present in the figure legend, table legend, main text, or Methods section.

n/a	Confirmed	
☐	☒	The exact sample size (n) for each experimental group/condition, given as a discrete number and unit of measurement
☐	☒	A statement on whether measurements were taken from distinct samples or whether the same sample was measured repeatedly
☐	☒	The statistical test(s) used AND whether they are one- or two-sided<br>*Only common tests should be described solely by name; describe more complex techniques in the Methods section.*
☐	☒	A description of all covariates tested
☐	☒	A description of any assumptions or corrections, such as tests of normality and adjustment for multiple comparisons
☐	☒	A full description of the statistical parameters including central tendency (e.g. means) or other basic estimates (e.g. regression coefficient) AND variation (e.g. standard deviation) or associated estimates of uncertainty (e.g. confidence intervals)
☐	☒	For null hypothesis testing, the test statistic (e.g. F, t, r) with confidence intervals, effect sizes, degrees of freedom and P value noted<br>*Give P values as exact values whenever suitable.*
☒	☐	For Bayesian analysis, information on the choice of priors and Markov chain Monte Carlo settings
☒	☐	For hierarchical and complex designs, identification of the appropriate level for tests and full reporting of outcomes
☒	☐	Estimates of effect sizes (e.g. Cohen's d, Pearson's r), indicating how they were calculated

Our web collection on statistics for biologists contains articles on many of the points above.

Software and code

Policy information about availability of computer code

Data collection	Data was collected through HTTP web portals using Firefox.
Data analysis	Analysis of the data was conducted using R version 4.2.2. Packages used include DESeq2 (v1.36.0), fgsea (v1.22.0), Seurat (v4.2.0), dplyr (v1.0.10), UCell (v2.0.1), ggsci (v2.9), speckle (v0.99.1), Giotto (v1.1.2), RANN (v2.6.1), reshape2 (v1.4.4). Custom code is available at https://github.com/alexcoulton/macrophage-atlas

For manuscripts utilizing custom algorithms or software that are central to the research but not yet described in published literature, software must be made available to editors and reviewers. We strongly encourage code deposition in a community repository (e.g. GitHub). See the Nature Portfolio guidelines for submitting code & software for further information.

Data

Policy information about availability of data

All manuscripts must include a data availability statement. This statement should provide the following information, where applicable:

- Accession codes, unique identifiers, or web links for publicly available datasets
- A description of any restrictions on data availability
- For clinical datasets or third party data, please ensure that the statement adheres to our policy

The scRNAseq atlas generated in this study has been deposited in Zenodo as a Seurat object under accession code 11222158 (https://doi.org/10.5281/zenodo.11222158). Source data are provided with this paper.

The atlas was composed of 32 studies (Azizi et al., 2018; Becker et al., 2022; Bi et al., 2021; Biermann et al., 2022; Borcherding et al., 2021; Braun et al., 2021; Chan et al., 2021; Che et al., 2021; S. Cheng et al., 2021; Durante et al., 2020; Jerby-Arnon et al., 2018; Khaliq et al., 2022; N. Kim et al., 2020; Krishna et al., 2021; Leader et al., 2021; Li et al., 2019; Lu et al., 2022; Maynard et al., 2020; Pelka et al., 2021; Pombo Antunes et al., 2021; Pu et al., 2021; Qian et al., 2020; Sharma et al., 2020; F. Wu et al., 2021; S. Z. Wu et al., 2021; Xu et al., 2021; Zhang et al., 2022; X. Zhang et al., 2021; Y. Zhang et al., 2021; Zheng et al., 2022; Zilionis et al., 2019)

The CPI data was obtained from 10 studies (Banchereau et al., 2021; Hugo et al., 2016; Kim et al., 2018; Liu et al., 2019; Mariathasan et al., 2018; McDermott et al., 2018; Miao et al., 2018; Patil et al., 2022; Riaz et al., 2017; Van Allen et al., 2015)

Research involving human participants, their data, or biological material

Policy information about studies with human participants or human data. See also policy information about sex, gender (identity/presentation), and sexual orientation and race, ethnicity and racism.

Reporting on sex and gender	We did not perform sex / gender specific analyses
Reporting on race, ethnicity, or other socially relevant groupings	We did not perform race / ethnicity specific analyses
Population characteristics	NA
Recruitment	NA
Ethics oversight	NA - This study was a third party reanalysis of existing data; all of the involved studies are cited in the manuscript

Note that full information on the approval of the study protocol must also be provided in the manuscript.

Field-specific reporting

Please select the one below that is the best fit for your research. If you are not sure, read the appropriate sections before making your selection.

[x] Life sciences [ ] Behavioural & social sciences [ ] Ecological, evolutionary & environmental sciences

For a reference copy of the document with all sections, see nature.com/documents/nr-reporting-summary-flat.pdf

Life sciences study design

All studies must disclose on these points even when the disclosure is negative.

Sample size	We attempted to compile the largest, most comprehensive dataset on tumour-associated macrophages to date, using publically available datasets.
Data exclusions	Studies without count data were excluded
Replication	We used robust statistical methods and validated usage of our atlas in two external datasets
Randomization	Data was collected from publicly available sources rather than generated de novo, and as such numbers in each group were determined by availability
Blinding	There was no specific blinding applied during analysis of the data

Reporting for specific materials, systems and methods

We require information from authors about some types of materials, experimental systems and methods used in many studies. Here, indicate whether each material, system or method listed is relevant to your study. If you are not sure if a list item applies to your research, read the appropriate section before selecting a response.

Materials & experimental systems

n/a	Involved in the study	
[x]	[ ]	Antibodies
[x]	[ ]	Eukaryotic cell lines
[x]	[ ]	Palaeontology and archaeology
[x]	[ ]	Animals and other organisms
[ ]	[x]	Clinical data
[x]	[ ]	Dual use research of concern
[x]	[ ]	Plants

Methods

n/a	Involved in the study	
[x]	[ ]	ChIP-seq
[x]	[ ]	Flow cytometry
[x]	[ ]	MRI-based neuroimaging

Clinical data

Policy information about clinical studies
All manuscripts should comply with the ICMJE guidelines for publication of clinical research and a completed CONSORT checklist must be included with all submissions.

Clinical trial registration	NA - Third party reanalysis of existing data
Study protocol	NA
Data collection	NA
Outcomes	NA

Plants

Seed stocks	NA
Novel plant genotypes	NA
Authentication	NA
